# Hybrid Imaging for Patient-Specific Dosimetry in Radionuclide Therapy

**DOI:** 10.3390/diagnostics5030296

**Published:** 2015-07-10

**Authors:** Michael Ljungberg, Katarina Sjögreen Gleisner

**Affiliations:** Department of Medical Radiation Physics, Lund University, 221 85 Lund, Sweden; E-Mail: katarina.sjogreen_gleisner@med.lu.se

**Keywords:** dosimetry, Monte Carlo, SPECT, hybrid, CT, absorbed dose, therapy, reconstruction, quantitation, activity

## Abstract

Radionuclide therapy aims to treat malignant diseases by systemic administration of radiopharmaceuticals, often using carrier molecules such as peptides and antibodies. The radionuclides used emit electrons or alpha particles as a consequence of radioactive decay, thus leading to local energy deposition. Administration to individual patients can be tailored with regards to the risk of toxicity in normal organs by using absorbed dose planning. The scintillation camera, employed in planar imaging or single-photon emission computed tomography (SPECT), generates images of the spatially and temporally varying activity distribution. Recent commercially available combined SPECT and computed tomography (CT) systems have dramatically increased the possibility of performing accurate dose planning by using the CT information in several steps of the dose-planning calculation chain. This paper discusses the dosimetry chain used for individual absorbed-dose planning and highlights the areas where hybrid imaging makes significant contributions.

## 1. Introduction

The increased diagnostic value of image fusion between function images from single-photon emission computed tomography (SPECT) or positron emission tomography (PET) combined with the anatomical information obtained from a computed tomography (CT) scan is unquestionable. This review focuses on the use of combined systems (mainly SPECT/CT) for radionuclide therapy applications rather than applications for diagnostic imaging. With access to an X-ray imaging unit (scout view as well as CT), improved absorbed dose calculations have become more widely available also for non-research clinical institutions since many of the early problems associated with the manual mathematical image registration of SPECT images and CT images, which were raised because acquisition involved acquisition on separate imaging systems, are now obsolete. 

### 1.1 Radiobiological Effects of Ionizing Radiation

The fundamental quantity for coupling the energy imparted by ionizing radiation interactions in tissue and the observed biological effects is the absorbed dose, which is defined as the quotient between the total energy deposited in a small volume and the mass of that volume. The unit is gray (Gy) which equals J/kg. In addition to the absorbed dose, there are other factors that affect the biological effect, such as the type of radiation, the radiosensitivity of the biological system, and the time pattern with which the absorbed dose is given. In diagnostic nuclear medicine, the levels of activity and the absorbed doses given to patients are comparably low, and the primary aim of radiation protection is to develop optimized procedures that maintain diagnostic accuracy while minimizing the risk of stochastic radiobiological effects [1]. In therapeutic nuclear medicine, higher levels of activity and absorbed doses are given with intent of controlling or eradicating tumours. The absorbed doses then need to be balanced to prevent the risk of inducing deterministic effects to healthy organs, *i.e.*, radiation-induced harmful tissue reactions [2]. Depending on the tissue irradiated, such effects may occur early or late after irradiation, and both the probability of occurrence and severity depends on the absorbed dose level. 

###  1.2 Clinical Protocols for Radionuclide Therapy

In contrast to diagnostic nuclear medicine, a characteristic for the radionuclides used in radionuclide therapy is the emission of particle radiation in their decay, such as electrons from β^−^ decay or α particles. Such particles have a short range in tissue and deposit their energy locally, thus giving a locally high absorbed dose in tissues where they are accumulated, such as tumours, while sparing surrounding tissues. Often, radionuclides that emit both particles and γ photons are preferable, where the γ photons can be used for imaging and thus image-based dosimetry. Another characteristic for the radionuclides used in therapy is that they have a longer half-life than those used in diagnostics, to maintain irradiation of tumours that generally have a long radiopharmaceutical retention. Comprehensive lists of therapeutic radionuclides and their decay properties can be found e.g., in references [3,4]. 

Radionuclide therapy has a long history, commencing in the 1940s with studies involving ^89^Sr, ^131^I, and ^32^P. The most widespread radionuclide therapy today uses ^131^I as sodium iodide for the diagnosis and treatment of benign thyroid conditions and thyroid cancer [5,6]. Clinically used radionuclides include, for instance, ^89^Sr, ^153^Sm, ^186^Re and ^223^Ra for the treatment of bone metastases [7,8], ^131^I-mIBG for the treatment of neuroblastoma and adult neuroendocrine tumours (NET) [9] and ^90^Y labelled to monoclonal antibodies for treatment of non-Hodgkins lymphoma [10]. Currently, new types of radionuclide therapies are being introduced, such as ^90^Y-labeled microspheres for intra-arterial treatments of tumours and metastases in the liver [11] and ^177^Lu- or ^90^Y-labeled peptides of NET [12,13].

Although dose planning is possible for many of the clinically used radionuclide therapies, the vast majority of treatments are still based on activity prescription using fixed amounts of administered activity or based on the patient weight or body surface. This can partly be explained by the lack of the required and comparably advanced dosimetry methods. Furthermore, the dominating side effect is hematologic toxicity due to irradiation of the bone marrow, which can be handled by clinical means to a large degree. The activity levels have also been established at amounts that are tolerated by a cohort of patients in clinical trials with the intent of avoiding side effects. However, given that the time that a radiopharmaceutical resides in the body varies between individual patients, the absorbed dose given to different patients also varies when prescribing based on a fixed activity level. If the treatment was instead tailored to the absorbed-dose profile of the individual patient, the administered activity could likely be increased for many patients with an expected increased treatment effect as a result.

For the currently emerging radiopharmaceuticals, such as ^90^Y-labeled microspheres and ^177^Lu- or ^90^Y-labeled peptides, the dominating toxicity is associated with late responding tissues, such as kidneys and liver, where the radiation-induced tissue reactions cannot be easily monitored during treatment. The absorbed dose for these organs can only be determined by means of image-based dosimetry, and thus the need for developing accurate dosimetry methods is increasing.

### 1.3 Examples of Radionuclide Therapies Based on Dose Planning

^131^I therapy of benign thyroid conditions is one of the few examples where absorbed dose planning is being routinely conducted in some countries. The principle of absorbed dose planning is to perform a pre-study using a tracer amount of activity prior to therapy. The uptake and retention of the ^131^I is then measured externally along with the mass of tissue that accumulates ^131^I. The absorbed dose obtained in response to the administered tracer activity is then calculated and expressed as a factor in units of Gy per megabecquerel (MBq). During therapy, this factor is used to determine the activity that needs to be administered to deliver a prescribed absorbed dose using the assumption of a linear relationship between the uptake pattern for the tracer and therapy administrations. Another approach for absorbed dose planning is being undertaken in neuroblastoma therapy using ^131^I-mIBG, where treatment is given in two separate activity administrations separated by a two-week interval [14]. By measuring the whole-body absorbed dose received from the first therapy administration, the activity for the second administration is calculated to obtain a total whole-body absorbed dose of 4 Gy for the two administrations. A similar approach was adopted in a multi-center clinical trial of ^177^Lu-Dotatate therapy of NET, which is currently being undertaken at the Skåne University Hospital in Lund, and Sahlgrenska University Hospital, Gothenburg, Sweden (EudraCT No. 2011-000240-16). The treatment is given in repeated administrations, each of 7.4 GBq, approximately two months apart. The number of such administrations given to individual patients is based on the kidney-absorbed dose, because the kidneys are regarded as the principal organs at risk. Thus, the kidney-absorbed dose is determined after each administration of ^177^Lu-Dotatate, and new administrations are given until the renal biologically effective dose (BED, Section 2.2) reaches 27 or 40 Gy, depending on the presence of additional risk factors [15].

## 2. Absorbed Dose Calculations

### 2.1. MIRD Calculation Scheme

The Medical Internal Radiation Dose (MIRD) scheme is a mathematical description of the transport of radiation energy from a source volume to a specific target volume [16,17]. The principles behind the MIRD-formalism remain the same from the organ level to the cellular level [18]. The most commonly used application of the MIRD formalism has been in diagnostic nuclear medicine where it is generally used for risk estimates of radiation-induced late stochastic effects but it is equally applicable for therapeutic situations if the source and target volumes can be accurately defined. The formalism is described in a simple form by the equation

(1) $$D_{r_{T}}={\tilde{A}}_{r_{S}}\cdot S_{r_{T}\leftarrow r_{S}},$$

where $D_{r_{T}}$ is the mean absorbed dose in a target volume, ${\tilde{A}}_{r_{S}}$ is the total number of disintegrations during a specific time interval (cumulated activity) in the source volume, and $S_{r_{T}\leftarrow r_{S}}$ is a factor describing the radiation transport, which is formally defined as the mean absorbed dose to the target volume $r_{T}$ per unit cumulated activity in the source volume $r_{S}$. The cumulated activity is calculated from

(2) $${\tilde{A}}_{r_{S}}=A_{o}\int_{0}^{\infty }f_{r_{S}}(t)dt,$$

where *A_o_* is the administered activity at time zero and *f*(*t*) is a time-dependent function that describes the uptake and washout of the radiopharmaceutical in the source organ. *f(t)* is often an exponential function or a combination of exponential functions. The $S_{r_{T}\leftarrow r_{S}}$ value can be described as

(3) $$S_{r_{T}\leftarrow r_{S}}=\frac{n\cdot E\cdot \phi (r_{T}\leftarrow r_{S})}{m_{r_{T}}},$$

where *n* and *E* are the number of particles emitted per nuclear transition and the energy of the particles, respectively, and $m_{r_{T}}$ is the mass of the target volume. The absorbed fraction, $\phi (r_{T}\leftarrow r_{S})$ defines the fraction of the energy emitted from the source $r_{S}$ that is absorbed in the target volume $r_{T}$. When broken down into its smallest constituents, Equation (1) becomes

(4) $$D_{r_{T}}=\frac{\sum_{r_{S}}\left({\tilde{A}}_{r_{S}}\cdot \sum_{i}n_{i}\cdot E_{i}\cdot \phi_{i}(r_{T}\leftarrow r_{S})\right)}{m_{r_{T}}}.$$

For practical purposes, in dosimetry for risk estimates within diagnostic nuclear medicine, the *S* values are pre-calculated using Monte Carlo calculations based on mathematical phantoms with organ dimensions defined from population reference data [19].

### 2.2. BED Calculations

When a cluster of cells is irradiated, different repair mechanisms are triggered. For many cell lines, the proportion of the cells that survive in response to an absorbed dose increases if sufficient time is given for repair. After short-duration irradiations, such as those in external beam radiotherapy, the majority of the repair takes place after the irradiation. In radionuclide therapy, where the time it takes to deliver the absorbed dose is based on the order of days, repair during irradiation becomes important. The radiobiological effects of irradiation thus depend on the absorbed dose as well as the absorbed-dose rate and the rate of cell repair. The linear-quadratic (LQ) model is today the most clinically used radiobiologic model. The underlying idea is that double strand breaks in the DNA molecule can result from interactions by one single particle, or two separate particle tracks. For the latter, if is there is a possibility that repair will take place between the two particle interactions when comparably low absorbed-dose rates are being used. The biological effective dose (BED) is a concept derived from the LQ model and is used to compare and estimate the biological effects in normal and malignant tissues for different time patterns of irradiation [20,21]. The proportion of cells that survive after irradiation is often described by cell-survival curves that represent the logarithm of the surviving fraction *SF* for different absorbed doses. In the LQ-model including the effects of repair, cell survival is described as

(5) $$ln(SF)=-[\text{α}D_{r_{T},\tau }+\text{β}G(\text{τ}){Dr_{T},\tau }^{2}],$$

where α and β are radiobiological parameters describing the rate of cell-lesion induction associated with one-particle and two-particle tracks, thus increasing linearly and quadratically with the absorbed dose, respectively. The parameter τ is the total time of radiation delivery, and $D_{r_{T},\tau }$ is the delivered absorbed dose to the target during time τ. The function *G* describes the increased cell survival due to repair, which thus affects the quadratic term only. This function is defined as the ratio of the radiobiological effect in the presence of repair in relation to that in the absence of repair and is given by

(6) $$G(\tau )=\frac{2}{D_{r_{T},\tau }^{2}}\int_{0}^{\tau }\overset{\text{i}}{D_{r_{T}}}(t)\left(\int_{0}^{t}{\overset{\text{i}}{D}}_{r_{T}}(w)\text{​}\omega (t-w)dw\right)dt,$$

where ${\overset{\text{i}}{D}}_{r_{T}}$ is the dose rate as a function of time *t* [22]. The function ω describes the repair of sub-lethal damage, which, for a single-phase exponential repair process with a rate of repair μ, is described as

(7) $$\text{ω}(t)=e^{-\mu t}.$$

When τ is short, such as in individual treatment fractions in external beam radiation therapy, the effect of the repair function ω(t) is small, and *G* tends to one. However, if τ is very long, the rate of repair becomes important in relation to the rate of lesion induction, which is proportional to the absorbed dose rate. The function *G* then tends to zero and the *SF* value in Equation (5) becomes completely governed by the linear term $\text{α}D_{r_{T},\tau }$

The BED is defined as

(8) $$BED=-\frac{\ln(SF)}{\text{α}},$$

which, following Equation (5), becomes

(9) $$BED=D_{\tau }\left[1+\frac{D_{r_{T},\tau }G(\tau )}{\alpha /\beta }\right]$$

The BED has attracted interest to describe the clinically observed radiation-induced reactions for late responding tissues in radionuclide therapy. For instance, Barone *et al.* [23] showed that kidney toxicity experienced during peptide receptor radiotherapy could be better explained in terms of BED instead of absorbed dose, and in [24] their dose-response data were compared with those of external beam radiotherapy with a good correspondence obtained. Furthermore, Strigari *et al.* [25] used BED in analysis of liver toxicity in treatment of liver tumours using ^90^Y-labeled microspheres. These and other evidence for dose-effect correlations in radionuclide therapy were recently reviewed [26].

## 3. The Scintillation Camera

Although PET systems are being increasingly used for diagnostic investigations, the scintillation camera is still the most commonly used device for measuring *in vivo* activity distributions in radionuclide therapy. The camera can be used in planar or SPECT mode and consists of a collimator, a scintillation crystal, and a positioning system. All cameras are connected to a computer for further image processing and analysis. There use of PET systems for application in radionuclide therapy imaging has been limited owing to a lack of suitable long-lived radionuclides. However, PET systems have recently been used to image ^90^Y-labeled micro-spheres [27] for the local treatment of metastases and tumours in the liver.

### 3.1. Principles

The principle behind imaging is that γ photons emitted in radioactive decays reach and interact with the scintillation crystal. The photon energy is thus transferred to electrons that further interact and excite atoms. In the de-excitation process, light with visible wavelengths is released and detected by photomultiplier tubes (PMTs) that act as amplifiers. The signal from these PMTs is used as a measure of the energy imparted by the gamma photons. By using a collimator, which is essentially a lead sheet with many parallel holes, photons with an oblique incidence angle are attenuated while those impinging more or less in a parallel direction are transmitted and interact in the NaI(Tl) crystal. With the signal from all (or many, depending on vendor) of the PMTs, the centroid coordinates of the signal are determined. These coordinates are then used to successively generate an image of where the photons have interacted. Using a parallel-hole collimator, the image is thus a projection showing where the radioactive decay occurs, *i.e.*, where the radiopharmaceutical is located.

### 3.2. Calibration

A scintillation camera provides an image of counts or, if normalized to the acquisition time, of the count rate (s^−1^). Owing to the properties of the parallel-hole collimator, it is possible to convert the count rate to activity (in MBq) by careful calibration. The system sensitivity, ε, (s^−1^·MBq^−1^) is an important parameter and needs to be determined for each combination of collimator and energy window. Calibration is performed by acquiring a planar image of a thin layer of a known amount of activity placed in air at a distance from the camera head. When using a parallel-hole collimator, the system sensitivity is independent of the position within the field of view, meaning that a single calibration factor can be used for all positions. For reconstructed SPECT images, the same calibration factor can be used for converting the recorded count rate in voxels to activity, provided that proper scaling with regards to the number of projection angles and the acquisition time per angle has been performed and that attenuation and scatter compensation have been applied. In principle, if the corrections are accurate, the corrected count rate represents the count rate that would have been detected if the source were located in air. Thus, the system sensitivity, as measured in air, can be applied for scaling from the voxel count rate to activity. However, the independence of distance for a parallel-hole collimator is only valid for the totally registered counts. The count distribution is blurred owing to the limited spatial resolution of the imaging system. Thus, the position of the counts is affected, which has the most severe effect on the measurement of small objects.

## 4. Physical Factors that Affect the Activity Measurement

**Figure 1:**
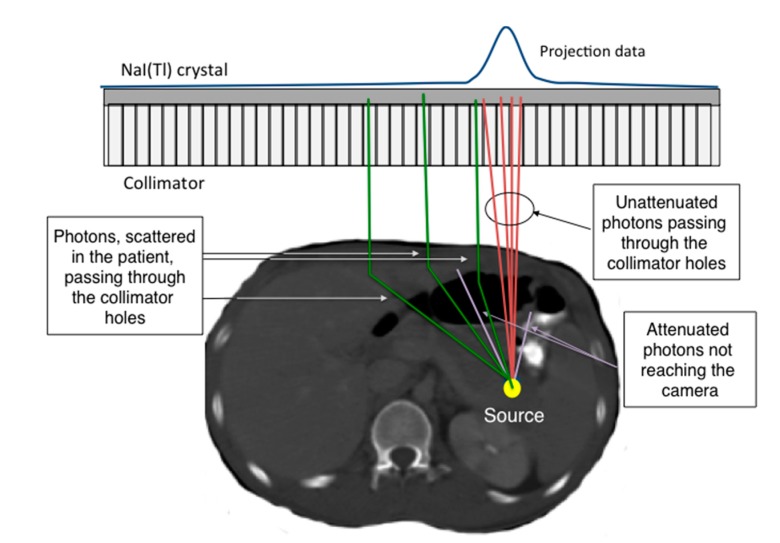
A schematic illustration of the different physical effects explained in the above text.

When measuring activity *in vivo*, several physical and camera-related effects will change the original fluence of photons. These effects make quantitative measurement impossible if proper compensation methods are not applied. Imaging protocols that are based on a single photon of energy, such as the 208 keV photons emitted from ^177^Lu, provide good opportunities to obtain a good image quality and accuracy in absorbed dose calculations. Imaging radionuclides that emit multiple photons of high energies, such as ^131^I, ^153^Sm, ^186^Re and ^223^Ra, or those that emit bremsstrahlung photons, such as ^90^Y, ^89^Sr and ^32^P, where the photon energies range continuously up to the maximum kinetic energy of the emitted β-particle require more sophisticated compensation methods. The following sections summarize the three major effects that are necessary to consider, as illustrated in Figure 1.

### 4.1. Photon Attenuation

Photon attenuation describes the reduction in detected photons due to interaction by photoelectric absorption or Compton scattering in matter. Interaction probabilities depend on the photon energy, the composition of the material, and the amount of material. In patient imaging, the attenuation of photons during their passage from the site of decay to the camera results in an image that contains fewer counts than would have been obtained if the activity were in air. Owing to the complex geometry and composition of a human, the amount of attenuation in SPECT projection measurements varies for different projection angles. If compensation is not applied, serious artefacts and false information may appear in the reconstructed SPECT images, which may lead to misinterpretations and false positives/negatives. In addition, for quantitative imaging, not applying attenuation correction yields severely underestimated values in the images.

### 4.2. Scatter Contribution

The term “scatter” refers to the fluence of secondary photons that have Compton-scattered in such a direction and with such small energy loss that they can pass through the collimator holes and be detected in the energy window. The underlying cause of scatter detection is the poor ability of the NaI(Tl) system to accurately measure the imparted energy. Owing to the random nature of the signal generation, there will be a fluctuation in the detected energy, even when the crystal is exposed to single photon energy, and the energy spectrum has a Gaussian-shaped distribution instead of a single peak. In order to register a sufficiently large number of counts, a comparably wide energy window is used. Therefore, photons that have scattered in the patient can pass through the energy window and thereby contribute to the image counts. However, scatter is an undesired contribution, since these photons have changed their initial direction and will thus not properly reflect the position of the decay. In diagnostic imaging, the main problem of scatter is a reduction in contrast. For quantitative imaging, scatter causes a false contribution of counts, which needs to be corrected.

### 4.3. Collimator Resolution

Parallel-hole collimators are constructed with narrow holes. However, there is a non-zero acceptance angle, and photons originating some distance away from the collimator surface with a non-parallel incidence angle will be able to pass through the holes. This results in uncertainty in the positioning of the site of decay, which increases as a function of distance. The system sensitivity remains the same, but the registered counts are spread over a larger detection area. As a result, images become blurry, as commonly characterized by the spatial resolution and the full-width at half-maximum (FWHM), which is approximately 1–2 cm. For quantification purposes, this blurring produces an underestimation of the activity concentration in small hot regions, since the counts originating from the region are spread over a larger volume. This effect is commonly termed as the partial volume effect (PVE) and thus reduces the possibility to accurately measure the activity concentration in small regions, typically with a diameter approximately three times the FWHM. Compensation for PVE using experimentally measured recovery coefficients is one simple approach and is often applied.

## 5. Planar-Image Based Activity Quantification

**Figure 2:**
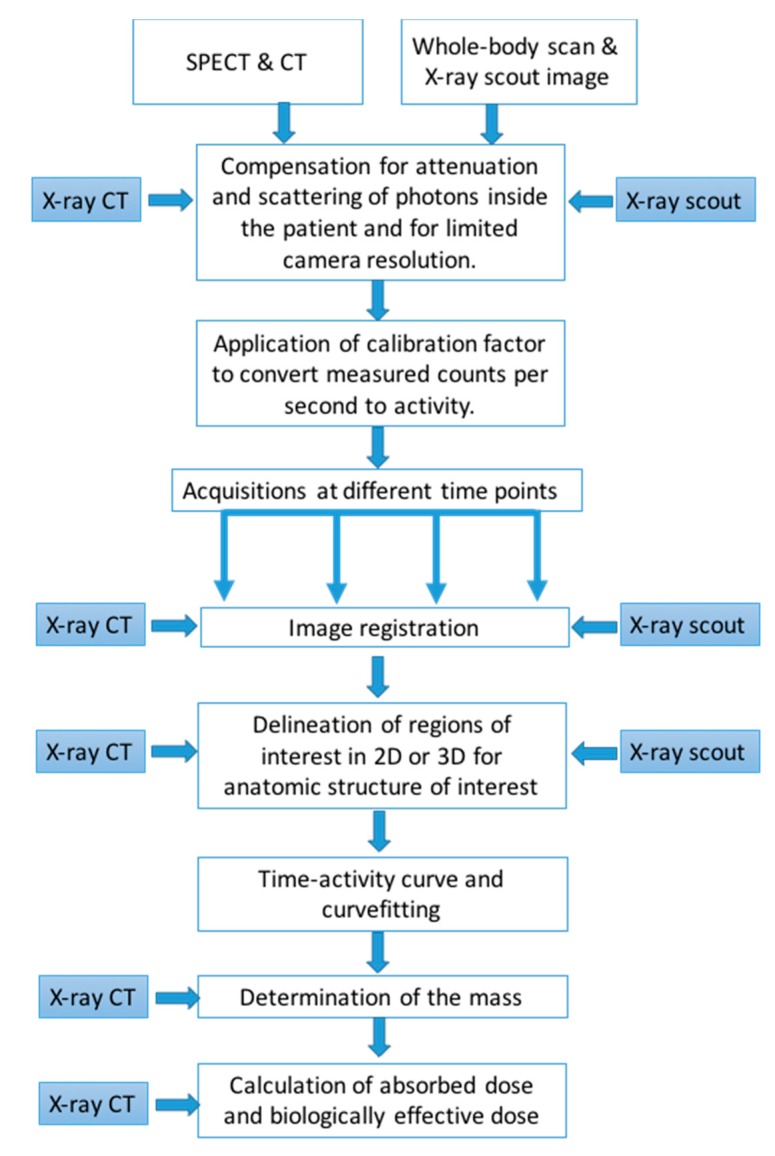
Flow-chart describing the different steps in the dosimetry chain and where information from 2D planar X-ray scout images or 3D tomographic CT images can be useful.

Figure 2 summarizes the steps required for accurate dosimetry procedures based on planar and SPECT images and highlights the steps where the anatomical information from X-ray imaging can be used.

### 5.1. Conjugate-View Principles

One of the first methods proposed for activity quantification using scintillation cameras was the planar conjugate-view technique [28]. The method is based on measuring an object in two opposite projection images. The difference in the detected count distribution in the two projections is then a function of the difference in photon attenuation. Generally, for attenuation compensation, the depth of the radioactive source in the patient needs to be known or estimated. However, if the geometrical-mean of the opposite projections is calculated, the source depth is cancelled out, and the attenuation correction factor becomes a function of the total thickness of the patient. We assume that *C*_0_ equals the count rate that would have been detected if the activity source were located in air, *C_a_* and *C_p_* are the detected count rates in the anterior and posterior view projections, respectively, *d* is the depth of the radioactive source along the anterior-posterior direction, µ is the linear attenuation coefficient for the tissues located along the photon’s trajectory, and *L* is the total thickness of the patient. Then, by applying the geometric mean, we obtain

(10) $$\sqrt{C_{a}C_{p}}=\sqrt{C_{0}e^{-\mu d}C_{0}e^{-\mu (L-d)}}=\sqrt{C_{0}^{2}e^{-\mu L}}=C_{0}\sqrt{e^{-\mu L}}$$

If a parallel collimator is used, then the system sensitivity, ε, is independent of the distance between the source and the collimator. The activity is then calculated using

(11) $$A\text{​}=\frac{\sqrt{C_{a}C_{p}e^{\mu L}}}{\varepsilon }$$

However, the independence of source depth is only valid for a point source. In clinically realistic situations, an additional correction factor for the source extension needs to be applied [29]. Moreover, in planar imaging, the activities located in organs and tissue, which superimpose in the anterior-posterior projection images, cannot be resolved, and corrections for background and overlapping tissues are required. The validity of such corrections, and the accuracy of the resulting activity value, should be carefully evaluated since they depend strongly on the activity and pharmacokinetics of the surrounding tissues.

### 5.2. Attenuation Correction Based on X-Ray Scout

Because the attenuation correction factor $e^{\mu L}$ in the conjugate-view method only depends on the total thickness of the patient and the attenuation coefficient, it is relatively straightforward to estimate it from a transmission measurement. Two acquisitions are required with the transmission source placed opposite to the camera head: one scan without the patient and another with the patient in position. By calculating the ratio of the image values in the two images, the attenuation correction factor is obtained on a pixel-by-pixel basis. One common solution is the use of a ^57^Co flood source as a transmission source. However, owing to the limited activity of such sources and the limited scan time, the derived attenuation maps suffer from noise.

**Figure 3:**
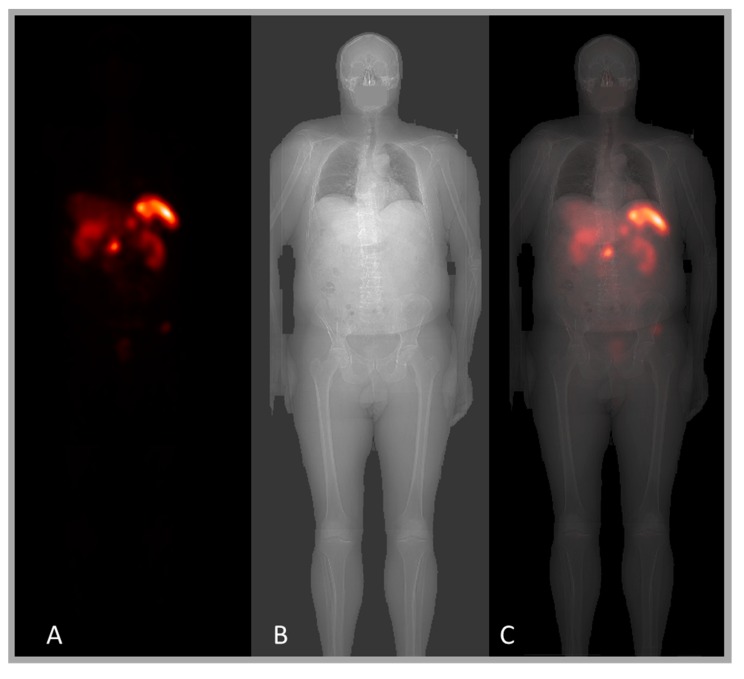
Patient whole-body images acquired during a ^177^Lu-Dotatate therapy. (**A**) is scintillation-camera image, (**B**) is an X-ray scout image, and (**C**) is an overlay of the scintillation-camera image on an X-ray scout image. The information from the scout image is useful for attenuation and scatter corrections, and for localization of the patient border and the lungs.

In modern CT systems, the axial scan length is normally determined by acquiring a scout overview prior to CT scanning. A scout image is essentially a planar X-ray investigation and is very well suited for use as an attenuation correction map in the conjugate-view method [30]. Its signal-to-noise ratio is high, the time required for acquisition is less than a minute, and the spatial resolution is exquisite. A potential problem for scout-based attenuation maps is the required scaling of the image values from the arbitrary units provided by the CT system to real attenuation values. Notably, this is a single scalar value, which, unfortunately, is often hidden in the CT system and may not be easily retrievable. If the scaling factor is not obtainable from the CT system, our group has developed an alternative scaling method, based on patient weight [31]. This method is currently being used in clinical patient studies at the Skane University Hospital. Figure 3 shows an example of a scintillation-camera whole-body image and the X-ray scout image of the same patient.

### 5.3. Scatter Compensation Based on X-Ray Scout

Different approaches exist for estimation of the scatter contribution in planar images, such as additional energy windows beside the photopeak energy window, methods based on build-up factors, effective attenuation coefficients, or deconvolution by scatter-point spread functions [32,33]. The latter approach requires information on the depth of the radioactive source in the patient as input. The location is often approximated as being along the central axis of the patient, and the required input information is then the total patient thickness. For instance, our group has developed a method based on deconvolution of whole-body scans by scatter-point spread functions. The latter are calculated using Monte Carlo simulations [34] of point sources located centrally in elliptically shaped water phantoms with different thicknesses. In application, the patient thickness over the abdominal region is determined from the scout image, the scatter-point-spread function for the corresponding phantom thickness is chosen from a database, and then deconvolution is applied.

### 5.4. ROI Definitions Using the X-Ray Scout

Delineation of regions of interests (ROIs) in planar images can be difficult since the boundaries between structures, which accumulate activity, are blurred by limited spatial resolution and superimposed activity from under- or overlying tissues. The X-ray scout is useful as a guide in the delineation of ROIs, especially for the delineation of the lung region and the contour of the patient body, which can be difficult to identify in the scintillation-camera scans.

## 6. SPECT Imaging

It is well known that planar-imaging-based activity quantification has limitations in dosimetry applications. The method is two-dimensional in nature, and the depth dimension is lost in the imaging process. Thus, the activities from different structures are superimposed in the images, and the masses of organs or tumours, which are needed for dosimetric estimates, cannot be estimated. Moreover, the *S* values cannot be determined in a patient-specific manner but need to be based on pre-calculated values. Therefore, tomographic SPECT imaging has advantages for therapeutic applications.

### 6.1. Acquisition of Multiple Projections

In SPECT, many projections are acquired around the patient along different directions. From the set of projections, tomographic images are reconstructed in which, if accurate corrections are applied, each voxel value reflects the activity located at a certain position in the patient. In the majority of applications, it is assumed that the activity is stationary during acquisition, and therefore the differences in the count-projection images depend only on the projection angle. Compared to planar whole-body imaging, SPECT has a limited axial field of view, which limits the anatomic region that can be covered by the scan. As a consequence, whole-body SPECT requires a longer acquisition time. Clinically, there are thus advantages and disadvantages with both methods; however, for accurate dosimetry in radionuclide therapy, the need for accuracy favours SPECT.

### 6.2. Tomographic Image Reconstruction

The objective of tomographic reconstruction is to solve the problem of determining the unknown activity distribution in the patient that produces the measured set of projection images [35]. Today, the most commonly used method is iterative reconstruction based on the maximum likelihood expectation maximisation (ML-EM). The underlying principle is that the imaging system is modelled in a forward projector step. Given an initial first guess of the radionuclide distribution, the forward projector calculates the image projections that are obtained in response to that distribution. By comparing calculated and measured projections, an error image is determined and used to update the initial estimate of the activity distribution. The procedure is repeated (iterated) until convergence has been reached *i.e.*, when the difference between the calculated and measured projections is regarded as being sufficiently small. An interesting benefit with this method is that, if physical effects such as photon attenuation, photon scatter, or effects of limited collimator resolution, are included in the forward projector step, then they will be intrinsically included in the estimation of the activity distribution in the patient, and thus attenuation compensation is achieved. The ordered-subsets expectation-maximisation (OS-EM) algorithm [36] is an alternative to the ML-EM method, where the difference mainly lies in how many projection angles are calculated before the error image is used to update the activity distribution. The benefit of OS-EM is that the reconstructed images reaches convergence much faster than the ML-EM algorithm and has therefore made iterative reconstruction clinically feasible.

### 6.3. MAP Reconstruction Using CT Information

It has been suggested that prior information about the object under reconstruction can be embedded into the reconstruction method. These so-called maximum *a posteriori* (MAP) [37] methods balance the strength of the error matrix in the update step. For example, if it is known that a radiopharmaceutical will distribute more or less homogeneously in an organ, then pronounced local variations between individual voxels are suppressed by a damping factor. This reduces the increase in noise that otherwise occurs for reconstructions using many iterations. Here, the CT image is useful since it provides anatomic information that can be used as a template, for instance if it is known *a priori* that a radiopharmaceutical does or does not accumulate in bony structures.

## 7. SPECT-Based Activity Quantification

### 7.1. Attenuation Correction Using CT Information

Attenuation correction requires information about the tissue distribution in the patient [38]. Earlier methods relied on transmission measurements using radionuclide sources mounted on the opposite side of the patient in relation to the camera [39]. However, the transmission information was of poor spatial resolution and had a low signal-to-noise ratio. With the introduction of SPECT/CT systems, a much better attenuation map can be obtained from CT, with improved accuracy and stability of the attenuation correction as a result. However, several steps need to be addressed before the CT images are useful, including: (a) conversion from CT Hounsfield numbers to attenuation coefficients for the photon energy of the SPECT radionuclide; (b) interpolation of the CT image to the SPECT voxel size and matrix size; (c) adjustment of the spatial resolution of the CT image to that of the SPECT system by low-pass filtering in order to avoid artefacts in SPECT images in regions with high signal gradients; and (d) ensuring that the CT and SPECT images are accurately co-registered. For the latter step, some vendor systems have had problems with patient couches that are too unstable and bend when moved between the CT and SPECT acquisition positions.

### 7.2. Scatter Correction Using CT Information

In many commercial systems, scatter correction is performed using additional energy windows defined beside the main photo-peak energy window [40,41]. The scatter is then estimated by scaling the recorded counts in these windows under the assumption that the counts represent the scatter distribution in the photo-peak window. The scatter can then be subtracted from the raw projection data or, more preferably, be implemented as a part in the reconstruction forward projector. These methods are relatively easy to implement but do not always reflect the true scatter distribution. An alternative is to analytically model the probability for photon scattering as part of the forward projection. Such modelling requires an estimate of the density distribution in the patient, which can be derived from CT images. The ESSE scatter model [42], developed by Frey and colleagues, is one example of such a method that has also been implemented by some commercial vendors. The advantage is that the modelled scatter component better reflects the distribution of the true scatter in the photon peak window, as compared to energy-window-based techniques.

### 7.3. Collimator Resolution Compensation

**Figure 4:**
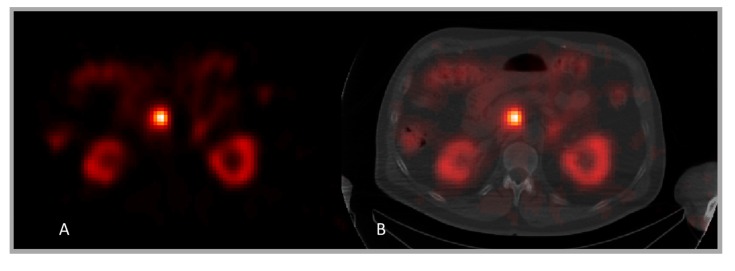
SPECT/CT image acquired during a ^177^Lu-Dotatate therapy, with activity localized in the kidneys, intestines and tumour. (**A**) is a SPECT image, while (**B**) shows SPECT overlaid on CT image. The CT image is useful for localization but also for attenuation and scatter corrections and for determination of the mass of the tissues where activity is localized.

Compensation for collimator resolution is being increasingly used to restore some of the blur caused by limited spatial resolution in SPECT imaging [43,44]. The basic idea is to model the effect of limited resolution as part of the reconstruction forward projection. Since the spatial resolution decreases with the distance between the site of decay and the camera, modelling is performed by convolution of the estimated activity distribution at different distances by point-spread functions with different widths. Usually, Gaussian functions with a width determined by the geometrical properties of the collimator are used. However, a drawback of this method is that it may create so-called Gibbs ringing in the images. Although the overall volume is restored, some wave-shaped artefacts can be seen, especially close to high-gradient regions such as edges. In addition, a decrease in the central region of objects can sometimes be seen, which, for tumours, can be falsely interpreted as a lower uptake. Compensations for collimator resolution do not generally use the CT information available in hybrid systems. Figure 4 shows an example SPECT/CT image. 

## 8. Image Registration

Image (co-)registration refers to the technique in which two images are brought into geometrical correspondence, so that the values in pixels (or voxels) with the same coordinates originate from identical anatomical positions in the patient. Image registration enhances the information from individual imaging modalities, such as scintillation-camera images and X-ray imaging, in 2D or 3D, since simultaneous anatomic and functional information is obtained. In radionuclide therapy, image registration can be applied to facilitate several steps in image-based dosimetry.

### 8.1. Registration of Planar Whole-Body Images and X-Ray Scout

For planar-based dosimetry, where whole-body images are acquired at multiple times after injection, for instance, at four time points over one week in ^177^Lu-Dotatate peptide receptor radiotherapy, image registration facilitates the image analysis, since an ROI delineated in one image can be directly transferred for analysis of the other images. If activity quantification is applied on the level of individual pixels [32] then image registration of the whole-body (WB) images and the attenuation map, as derived from the X-ray scout image, is necessary. In addition, such registration makes it possible to use the X-ray scout as guide in the delineation of ROIs [31]. Although care is taken to reproduce the patient position under the camera using devices such as positioning lasers, the body position becomes quite different for different acquisition occasions. To transform the images geometrically, a non-rigid transformation is necessary and has been implemented in a method tailored for whole-body image registration [45].

### 8.2. Registration of Sequential SPECT/CT Images

In modern SPECT/CT systems, registered images are normally provided by the camera workstation. Most of the time, this is accomplished by interpolation of the CT image to the matrix dimensions of the SPECT image, so that they can be shown in overlay and used for corrections. The interpolation uses information in the image headers for its completion, such as the matrix dimensions, the voxel spacing, and the coordinates of the image centre in 3D. When implementing such interpolation, it is important that the normalisation of image values is performed correctly. Otherwise, image values may be affected. This interpolation thus provides the registered SPECT and CT images, which are necessary for proper corrections for attenuation and scatter in the tomographic reconstruction.

For dosimetry in radionuclide therapy, where imaging is performed at multiple times, the drawing of volumes of interest (VOIs) in 3D is a time-consuming operation, and image registration then offers the advantage of enabling a transferal of VOIs delineated in one data set to the other data sets. The SPECT image information is difficult to use as basis for determining the geometric transformation describing the patient movement between scans, since the radiopharmaceutical is redistributed over time. CT images are better suited for this task, and, since they are acquired in conjunction to each SPECT study, once registration of the CT images from the different time points has been made, the same geometric transformation can be applied for spatial transformation of the sequence of SPECT images [46].

## 9. Combined SPECT/Whole Body Imaging

In situations when multiple SPECT/CT imaging is not possible or the axial FOV obtained from WB imaging is desired, a combined SPECT/WB approach can be adopted. Here, WB imaging is performed for all time points, and images are processed using the geometric-mean method, including compensation for scatter, attenuation, over-lapping activity, and background. On one occasion, a quantitative SPECT/CT study is performed in conjunction with the WB study. The time-activity data obtained from the WB images for each organ of interest are used to determine the time-retention curve. The amplitude of this curve is then renormalized so that the curve passes through the organ activity as determined from SPECT/CT [47]. The underlying assumption is thus that the SPECT/CT quantitative data are more accurate than the planar-derived activity data.

## 10. Time-Activity Curve

Imaging for dosimetry in radionuclide therapy applications is normally performed during the first week after injection. At best, 6–7 acquisitions can be obtained, but more often 3–4 times are considered clinically manageable. Using this sparsely sampled time-activity curve (TAC), the objective is to describe the pharmacokinetics of the radiopharmaceutical during the total time that it resides in an organ. The time integral of the TAC until infinity is equal to the number of decays that occur in the organ, *i.e.*, the cumulated activity, from which the absorbed dose is calculated (Equation (2)). The activity change as a function of time depends both on the biological behaviour of the radiopharmaceutical, *i.e.*, the uptake and washout, and the decreasing number of radioactive nuclei due to decay. Since many biological processes follow exponential patterns, different combinations of exponential functions are often used and fitted to the time-activity data. The TAC beyond the last measurement can be estimated from the fitted curve from the first week by extrapolation to infinity.

## 11. Voxel-Based Absorbed Dose Calculation

### 11.1. The Monte Carlo Method

**Figure 5:**
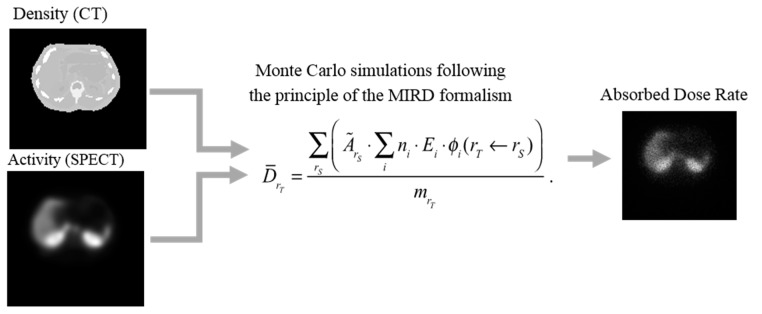
The principles for voxel-based absorbed dose calculations from a set of quantitative SPECT images in units of Bq and a set of anatomical CT images in units of g·cm^−3^. The Monte Carlo method calculate the radiation transport from each source voxel (*r_S_*) to all other target voxels (*r_T_*) from the principles described in Equation (4) by explicit simulate the particle tracks of photons and charge-particles.

The Monte Carlo method can be used to model tracks of individual particles (photons, electrons, protons, *etc.*) and their energy deposition pattern [48]. The energy deposited in different sites of interaction is analysed to estimate the absorbed dose distribution. Monte-Carlo-based dosimetry is regarded as the most accurate method and can, when combined with CT information, be used for patient-specific estimations [49,50,51]. Especially in regions were the density and tissue composition vary, such as lung/tissue and tissue/bone boundaries, the Monte Carlo method provides better results than other methods. Practically, a quantitative SPECT image is used as an estimate of the source distribution, *i.e.*, that of the radioactive radiopharmaceutical. From each voxel coordinate, representing a location within the patient, charged particles and photons are emitted according to the decay scheme for the radionuclide by assuming that the SPECT-image value in the particular voxel represents the activity in that location. The particles are traced through the CT volume by sampling from appropriate interaction types, and the deposited energy is stored along the track. The result is a 3D map of the absorbed dose rate (Figure 5).

### 11.2. Patient Geometry from CT Information

Monte-Carlo-based absorbed dose calculations are based on simulating the individual tracks of particles and photons, scoring the energy deposition along the tracks. Such simulations rely on accurate estimates of mass-attenuation coefficients and stopping-power values for the energies of the particles and photons, which in turn depend on the mass density and tissue composition. The density distribution can be estimated from a CT scan of the patient. Earlier, when using stand-alone SPECT systems, it was necessary to use CT images from other systems and software-based image registration. With the advent of hybrid SPECT/CT systems, registered CT images are obtained directly. It is, however, necessary to convert the CT image values, in Hounsfield numbers, to mass density values using calibration measurements. Moreover, it is important to be aware of artefacts in the CT images due to metal implants and contrast agents, which may introduce errors in the absorbed dose calculation.

## 12. Pre-Clinical Dosimetry with μSPECT/μCT and μPET/μCT

**Figure 6:**
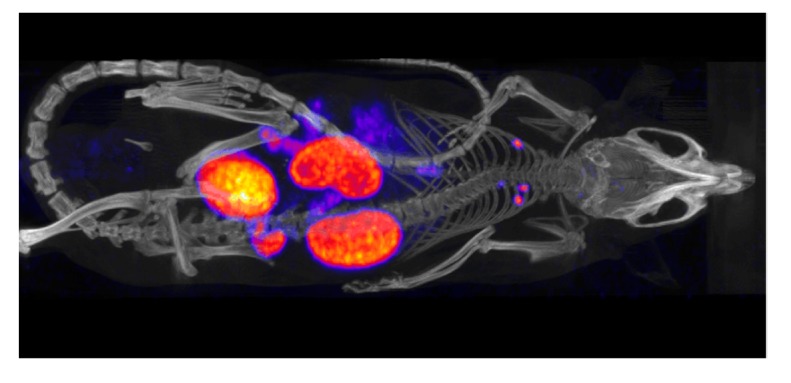
An example of a pre-clinical mouse study shown as fused images obtained from a combined μSPECT/μCT system. The mouse was injected with a ^99m^Tc-labelled peptide mainly excreted through the kidneys. (Courtesy—Thuy Tran, Lund Biomedical Imaging Center, Lund, Sweden).

Developments of new radiopharmaceuticals for human use are generally preceded by pre-clinical studies, where the biokinetic properties are studied in detail. In principle, preclinical imaging is governed by the same principles as clinical imaging; however, the requirement for better spatial resolution and smaller fields of view has driven the development of dedicated pre-clinical systems. These include high-resolution SPECT and PET systems in combination with high-resolution CT systems, which have been commercially available for the last 10–15 years. The spatial resolution is superior compared to that of clinical systems, and, for SPECT, the system sensitivity is higher since it is possible to use multiple detectors in combination with pinhole collimators. Figure 6 shows an example of a pre-clinical SPECT study of a mouse with fused CT information.

## 13. Conclusion

The anatomical information obtained either from planar scout images or from high-resolution tomographic CT images is not only useful for image fusion and localization, but also in many of the steps necessary for an accurate absorbed dose calculation in image-based radionuclide therapy dosimetry.
